# Neutrophil elastase promotes low molecular weight cyclin E1 formation to accelerate osteosarcoma proliferation

**DOI:** 10.3389/fimmu.2025.1647913

**Published:** 2025-09-01

**Authors:** Jiuhui Xu, Qianyu Shi, Fanwei Zeng, Tingting Ren, Ran Wei, Xiaodong Tang

**Affiliations:** ^1^ Department of Musculoskeletal Tumor, Peking University People’s Hospital, Beijing, China; ^2^ Beijing Key Laboratory of Musculoskeletal Tumor, Peking University People’s Hospital, Beijing, China

**Keywords:** cyclin E1, NETs, neutrophil, neutrophil elastase/ELA2, osteosarcoma

## Abstract

**Introduction:**

Osteosarcoma (OS) is the most common primary bone malignancy, characterized by aggressive local invasion and a high propensity for metastasis. We previously reported that cyclin E1 is upregulated in osteosarcoma. In this study, we identified a cytoplasmic, low molecular weight cyclin E1 isoform (LMW-cyclin E1) in osteosarcoma that is significantly associated with poor patient outcomes.

**Methods:**

We collected RNA sequencing data to analyze the cyclin E1 (*CCNE1*) expression and performed Western blot assay, immunofluorescence, and immunohistochemistry staining to validate cyclin E1 expression in OS. We also analyzed the correlation between its expression levels and the overall and progression-free survival rates of patients with OS. Small interfering RNA and plasmids were constructed to regulate neutrophil elastase (ELA2) expression to explore the mechanism of low molecular weight cyclin E1 formation in OS. Neutrophils isolated from healthy donors were cocultured with OS cells to test the function of ELA2, and its effect was further validated in BALB/c mice. The relationship between neutrophil infiltration and OS progression was analyzed in 34 primary OS tissues and 33 OS lung metastasis tissues.

**Results and discussion:**

Mechanistically, we found that ELA2, primarily derived from tumor-associated neutrophils, cleaves full-length cyclin E1 to generate LMW-cyclin E1, which accelerates OS proliferation. Moreover, neutrophil infiltration was associated with OS lung metastasis. OS cells also induced neutrophil extracellular trap formation, which further amplified ELA2 release. Depleting neutrophils or inhibiting ELA2 significantly suppressed OS malignancy. Hence, targeting neutrophil–osteosarcoma crosstalk may be a potential novel therapeutic strategy.

## Introduction

Osteosarcoma (OS) is the most frequent primary solid malignancy of bone in children and adolescents, with an annual incidence of 6.7 per million (1). The most common primary sites of OS are the metaphyses of long bones, especially the distal femur, the proximal tibia, and the proximal humerus (2). The current standard therapeutic strategy for OS is surgical resection alongside neoadjuvant and adjuvant chemotherapy. However, the 3- and 5-year overall survival rates remain 79% and 71%, respectively, and have plateaued for four decades (1, 3). Hence, novel mechanisms and therapeutic strategies for OS are urgently required to improve the patients’ prognoses.

Cyclin E1 is a regulatory subunit of cyclin-dependent kinase 2 (CDK2). The cyclin E1–CDK2 complex can regulate S-phase-specific genes, which are vital in the G1/S phase transition by enhancing DNA replication (4). Cyclin E1 participates in many aspects of tumorigenesis, and dysregulation of cyclin E1 has been documented in ovarian cancers, breast cancer, and many other cancer types (5, 6). In recent years, a low molecular weight cyclin E1 (LMW-cyclin E1) isoform has been detected in several types of tumors. LMW-cyclin E1 was reported as the more hyperactive isoform compared to full-length cyclin E1 (FL-cyclin E1). LMW-cyclin E1 has been shown to bind more efficiently to CDK2 and to mediate multiple biological processes in tumor progression, such as drug resistance and tumor metastasis (7–10). We have previously reported that cyclin E1 is significantly overexpressed in osteosarcoma, that its expression is correlated with osteosarcoma progression, and that it can serve as a prognostic biomarker for osteosarcoma (11). However, whether the LMW-cyclin E1 isoform exists in OS remains uncertain and needs to be further explored.

Neutrophils are the most abundant immune effector cells, accounting for 50%–70% of all leukocytes (12). Previously, neutrophils were thought to be involved only in the internal inflammatory response, and their remarkable roles in tumor progression were overlooked because of their incapacity for proliferation and limited half‐life. Recent research has shed light on neutrophils’ actions in solid tumors (13, 14). In osteosarcoma, an increase in the neutrophil-to-lymphocyte ratio (NLR) during treatment has been reported to be significantly associated with shorter median overall survival, indicating that neutrophils play a protumor role in OS progression (15). However, the function of neutrophils in OS remains unclear and unvalidated. Neutrophil extracellular traps (NETs) are structures composed of granule proteins and decondensed chromatin, which are the expression patterns of neutrophils in response to specific stimuli (16). Neutrophil elastase, a serine protease expressed in primary neutrophils, is associated with many pathophysiological processes, such as anti-infection, activation of dormant cancer cells, and remodeling the tumor microenvironment (17).

In this study, we aimed to determine whether LMW-cyclin E1 is present in OS cells and tissues and to investigate the biological mechanisms regulating LMW-cyclin E1 formation.

## Materials and methods

### Cell culture

143B (CVCL_2270), KHOS (CVCL_2546), HOS (CVCL_0312), MG63 (CVCL_0426), U2OS (CVCL_0042), and SaoS2 (CVCL_0548) cells were human osteosarcoma cell lines, and K7M2 cells were mouse osteosarcoma cell lines; all were purchased from the American Type Culture Collection. The human normal osteoblastic hFOB 1.19 cell line was kindly provided by the Department of Hematology, Peking University People’s Hospital. 143B, U2OS, SaoS2, K7M2, and hFOB 1.19 cells were cultured in DMEM (Gibco, American); KHOS and MG63 cells were cultured in RPMI-1640 (Gibco); and HOS cells were cultured in MEM (Gibco), each supplemented with 10% fetal bovine serum (FBS, Wisent, Canada) and 1% penicillin/streptomycin (Gibco). The cells were all cultured in an incubator at 37°C in humidified air with 5% CO_2_. All human cell lines were authenticated using STR (or SNP) profiling within the last 3 years, and all experiments were performed with mycoplasma-free cells.

### RNA sequencing data from a public database

The RNA sequencing data of osteosarcoma tissues were obtained from the Therapeutically Applicable Research to Generate Effective Treatments on Osteosarcoma (TARGET‐OS, phs000468) at https://portal.gdc.cancer.gov/projects/TARGET‐OS. The gene expression of CCNE1 was analyzed to investigate its correlation with patients’ prognosis.

### Human osteosarcoma tissues

OS samples were collected from Peking University People’s Hospital, with approval from the Ethics Committee of Peking University People’s Hospital. Informed consent was obtained from all patients. The samples to detect cyclin E1 expression were from 69 primary osteosarcoma *in situ* cases, and the samples used to detect neutrophil infiltration and ELA2 expression were from 34 primary osteosarcoma tissues and 33 lung metastasis lesions, which were processed as a tumor microarray. The tissues were fixed with 4% paraformaldehyde and made into a tissue microarray as described previously (18).

### Mouse experiments

The animal experiments were approved by the Ethics Committee of Peking University People’s Hospital (2023PHE123) and conducted according to the guidelines for the care and use of laboratory animals.

Four-week-old BALB/c mice were purchased from Beijing Vital River, China to establish the mouse OS model. The BALB/c mice were randomly divided into two groups: 1 × 10^7^ K7M2-lvNC cells and K7M2-lvELA2 were subcutaneously injected into the right side of the mice individually. After 25 days, the mice were killed, and the tumors were isolated for further analysis. For neutrophil exhaustion, 1 × 10^7^ K7M2 cells were subcutaneously injected into the right side of the mice. The mice were divided into two equal groups and treated with anti-Ly6G (127649, BioLegend, United States) and anti-IgG (50 μg/head, intraperitoneal injection, I4131, Sigma-Aldrich, United States) every 4 days. After 25 days, the mice were killed, and the tumors were isolated for further analysis.

### Co-immunoprecipitation assay and Western blot

Cultured cells were lysed with a mixture of lysis buffer (9803, Cell Signaling Technology, United States), protease inhibitor (B14001, Selleck), and phosphatase inhibitor (B15001, Selleck, United States). The protein concentration was measured using the BCA protein assay kit according to the manufacturer’s protocol (PC0020, Solarbio, China). The whole-cell lysate protein dilution was incubated with cylcin E1 antibody (1:50) overnight at 4°C. Protein A/G agarose beads (sc-2003, Santa Cruz Biotechnology, United States) were washed twice with cold PBS and added to the antigen–antibody dilution for another 1.5-h incubation at 4°C. The sample was centrifuged to collect the beads–antigen–antibody complex.

For Western blot, the proteins were loaded into 10% SDS-PAGE gels for protein separation. Protein bands were incubated with specific primary antibodies, including cyclin E1 (1:500), calpain 1 (1:1,000), calpain 2 (1:1,000), ELA2 (1:300), RB (1:1,000), pRB (1:500), and GAPDH (1:2,000). Proteins were visualized using Image Lab Software (Bio-Rad, United States), and the protein bands were quantitatively analyzed using ImageJ software. Information on the antibodies is provided in **Supplementary Table S3**.

### Neutrophil isolation and flow cytometry validation

Neutrophils were isolated from the peripheral blood of healthy donors. Peripheral blood was mixed in equal volume with erythrocyte sedimentation solution (R1000, Solarbio) and set at room temperature for 30 min. The neutrophils were isolated by density gradient separation using lymphocyte separation medium (LTS1077-1) and centrifuging at 500 × *g* for 25 min at room temperature. Neutrophils were confirmed to be of > 90% purity by flow cytometric analysis with antihuman CD66b Antibody (E-AB-F1267E, Yojanbio, China).

### Co-culture system for neutrophils and osteosarcoma cells

A 0.4-μm transwell plate (LabSelect) was used to construct the noncontact coculture system. Neutrophils were isolated as previously mentioned; 5 × 10^5^ osteosarcoma cells were seeded into the lower layer of the transwell plate overnight. Subsequently, 5 × 10^5^ neutrophils were added to the upper layer for a 24-h coculture. For the contact co-culture system, 5 × 10^5^ osteosarcoma cells were preseeded into a six-well plate, and 5 × 10^5^ neutrophils were added directly to the same plate.

### Immunofluorescence staining and immunohistochemistry staining

Anti-ELA2 antibody (1:50), anticyclin E1 antibody (1:100), and antihistone H3 antibody (1:100) were used for cell immunofluorescence. Rhodamine Phalloidin (R415, Invitrogen, United States) was used to visualize F-actin in OS cells. Anti-ELA2 antibody (1:400), anti-CD66b antibody (1:2,000), and anti-Ki67 (1:4,000) were used for immunohistochemistry (IHC) staining.

### Giemsa staining

Neutrophils collected from healthy donors were stained using the Fast Giemsa Stain Kit (40751ES02, Yeasen, China) according to the manufacturer’s protocol. The images were obtained and observed under a microscope (Leica, Germany).

### RNA extraction and qPCR assay

The cells were lysed using TRIzol (Invitrogen), and total RNA was isolated with isopropanol and 75% ethanol. PrimeScript RT Master Mix (TaKaRa Biotechnology, Japan) was used for reverse transcription. qPCR was performed to measure the relative expression levels of target genes using SYBR Green premix Ex Taq (TaKaRa, RR420A). The primers are all listed in **Supplementary Table S1**. The relative fold changes in gene expressions were calculated using the 2^−▵▵^Ct method.

### CCK8 and colony formation assay

For the CCK8 assay, 3 × 10^3^ 143B or U2OS cells were plated into a 96-well plate. To measure the 24-h IC_50_ values of AZD 9668 (Cayman, United States), different concentrations of AZD 9668 were added to the cell culture medium. Cell Counting Kit-8 (CCK8; Dojindo , Japan) was added to the culture medium (1:10). After 1 h of incubation, the optical density (OD) value was measured at 450 nm using a microplate reader (Bio-Rad).

### SiRNA and plasmid transfection

SiRNA targeted calpain 1, calpain 2, negative control siRNA, and ELA2 overexpression plasmids were designed and synthesized by Hanbio Tech (Shanghai, China), with the sequences listed in **Supplementary Table S2**. The 143B and U2OS cells were transfected with Lipofectamine 3000 transfection reagent (L3000015, Thermo Scientific, United States).

To generate stable ELA2 overexpressed K7M2 cells, K7M2 cells were infected with 10 multiplicity of infection (MOI) lentivirus (Lv), followed by 3 mg/ml puromycin selection for 1 week.

### Enzyme-linked immunosorbent assay

The cell culture supernatants were collected and centrifuged to remove cellular fragments, and then used immediately or stored at −80°C. Enzyme-linked immunosorbent assay (ELISA) was performed according to the manufacturer’s instructions, using an enzyme‐linked immunosorbent assay kit (CSB-EL007587HU, CUSABIO, China) for ELA2 and an ELISA assay kit (RX102517H, Rruixin Bio, China) for NETs.

### Cell cycle assay

The cell cycle assay was performed using PI staining, and the cell cycle and apoptosis analysis kit (C1052, Beyotime, China) was used according to the operator’s manual. Briefly, the cells were washed with cold PBS and mixed with 70% ethanol for 2 h. PI stain buffer and RNase A were added and incubated at 37°C for 30 min. The cells were then subjected to further flow cytometry.

### Statistical analyses

Data were expressed as mean ± standard deviation (SD). An unpaired two-tailed Student’s *t*-test was used for the analysis of two groups. One-way ANOVA was used for comparisons among several different groups. The relation between neutrophil infiltration and osteosarcoma lung metastasis was analyzed using Fisher’s exact test. Chi-square test was performed to analyze the distribution of ELA2 expression among different neutrophil infiltration groups. The relationship between gene expression and patient survival was assessed using Kaplan–Meier survival analysis. All statistical analyses were performed with the GraphPad Prism 9.0. The level of significance was chosen as *p* < 0.05.

## Results

### A special low molecular weight cyclin E1 isoform was present in osteosarcoma cell lines and tissues

**Figure 1:**
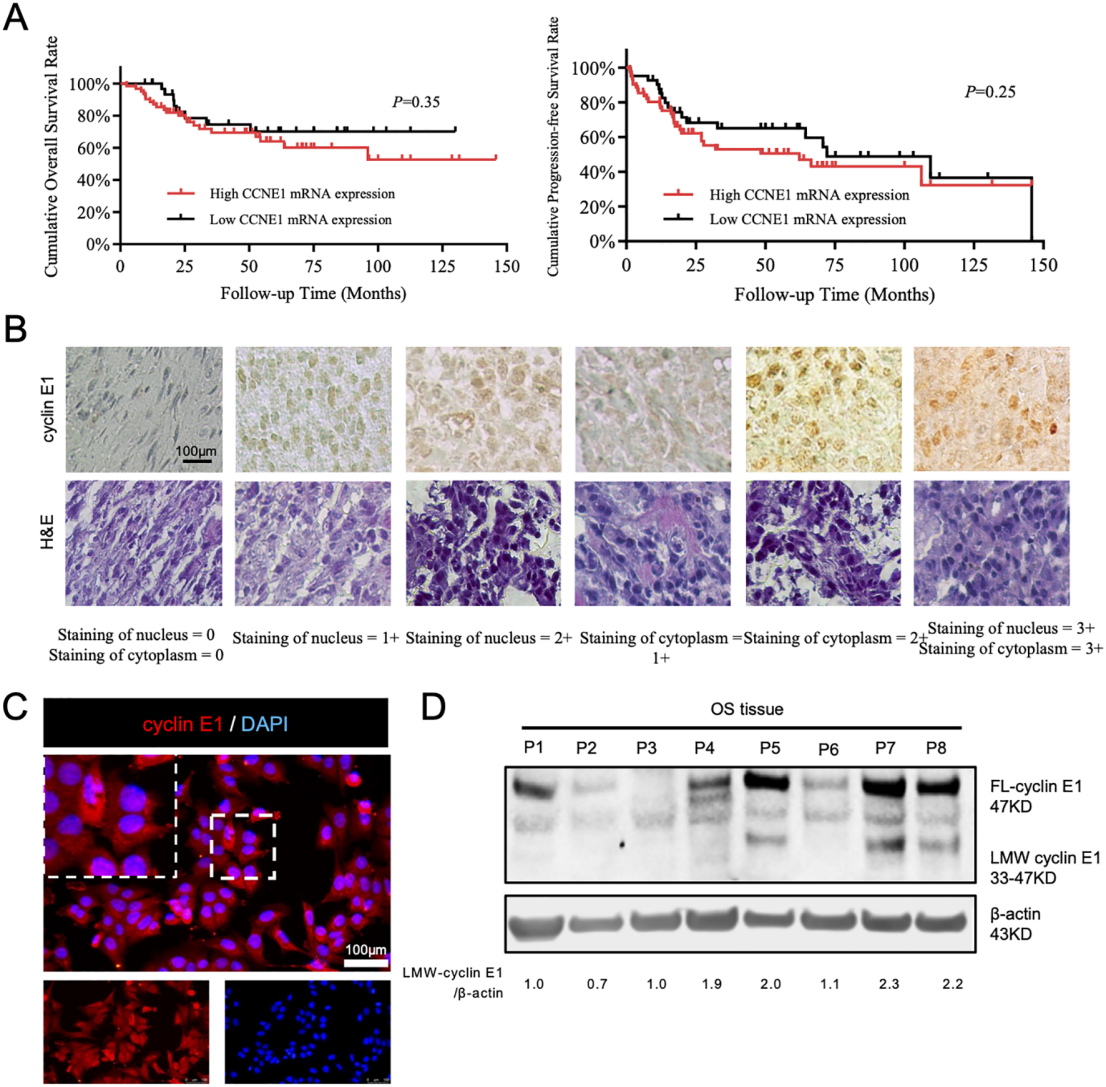
A special subtype of cyclin E1 was expressed in osteosarcoma cells and tissues. **(A)** Correlation analysis between CCNE1 mRNA expression and overall survival rate and progression-free survival rate (Kaplan–Meier survival analysis). **(B)** Immunohistochemical staining assay to detect cyclin E1 expression in an osteosarcoma tissue microarray, showing that cyclin E1 was distributed in both the cellular nucleus and cytoplasm of osteosarcoma tissue (*N* = 69; all samples were from osteosarcoma *in situ*; scale bar: 100 μm). **(C)** Immunofluorescence staining results showed that cyclin E1 was expressed in the cellular nucleus and cytoplasm (red: cyclin E1; blue: DAPI; scale bar: 100 μm). **(D)** Western blot analysis showed FL-cyclin E1 and LMW-cyclin E1 in osteosarcoma tissues. The FL-cyclin E1 protein was 47 kDa, and the LMW-cyclin E1 protein bands ranged from 33 to 47 kDa. Reproduced with permission of reference 11, our previously published article. The license number is 6094190822325, and the license was uploaded in the supplementary files. (*N* = 8; the assay was replicated three times).

As we previously reported, cyclin E1 and its coding gene, *CCNE1*, were both highly expressed in OS tissues. The expression of cyclin E1 protein was significantly correlated with overall survival, progression-free survival, and sensitivity to neoadjuvant chemotherapy (11). However, CCNE1 mRNA expression was not associated with patients’ prognosis via analyzing the mRNA data from public databases (**Figure 1A**), which suggests there may be a special post-transcriptional regulation mechanism for cyclin E1 in osteosarcoma.

To further investigate the cyclin E1 regulation mechanism, we first used immunofluorescence and immunohistochemistry staining to analyze cyclin E1 expression and distribution in osteosarcoma cells and 69 osteosarcoma lesions *in situ*. The staining results showed that cyclin E1 was located in both the osteosarcoma cellular nucleus and cellular cytoplasm, whereas it was located only in the cellular nucleus of the human normal osteoblastic hFOB 1.19 cell line, which indicated that different cyclin E1 isoforms with individual distributions existed in osteosarcoma (**Figures 1B, C**; **Supplementary Figure S1**). The expression of cyclin E1 in osteosarcoma samples was further investigated using a Western blot assay, and the results revealed that, in addition to FL-cyclin E1 in OS, which was shown at 47 kDa, several distinct low molecular weight protein bands were also observed at 33–47 kDa (**Figure 1D**).

### LMW-cyclin E1 was located in the cellular cytoplasm and related to osteosarcoma patients’ poor outcome

**Figure 2:**
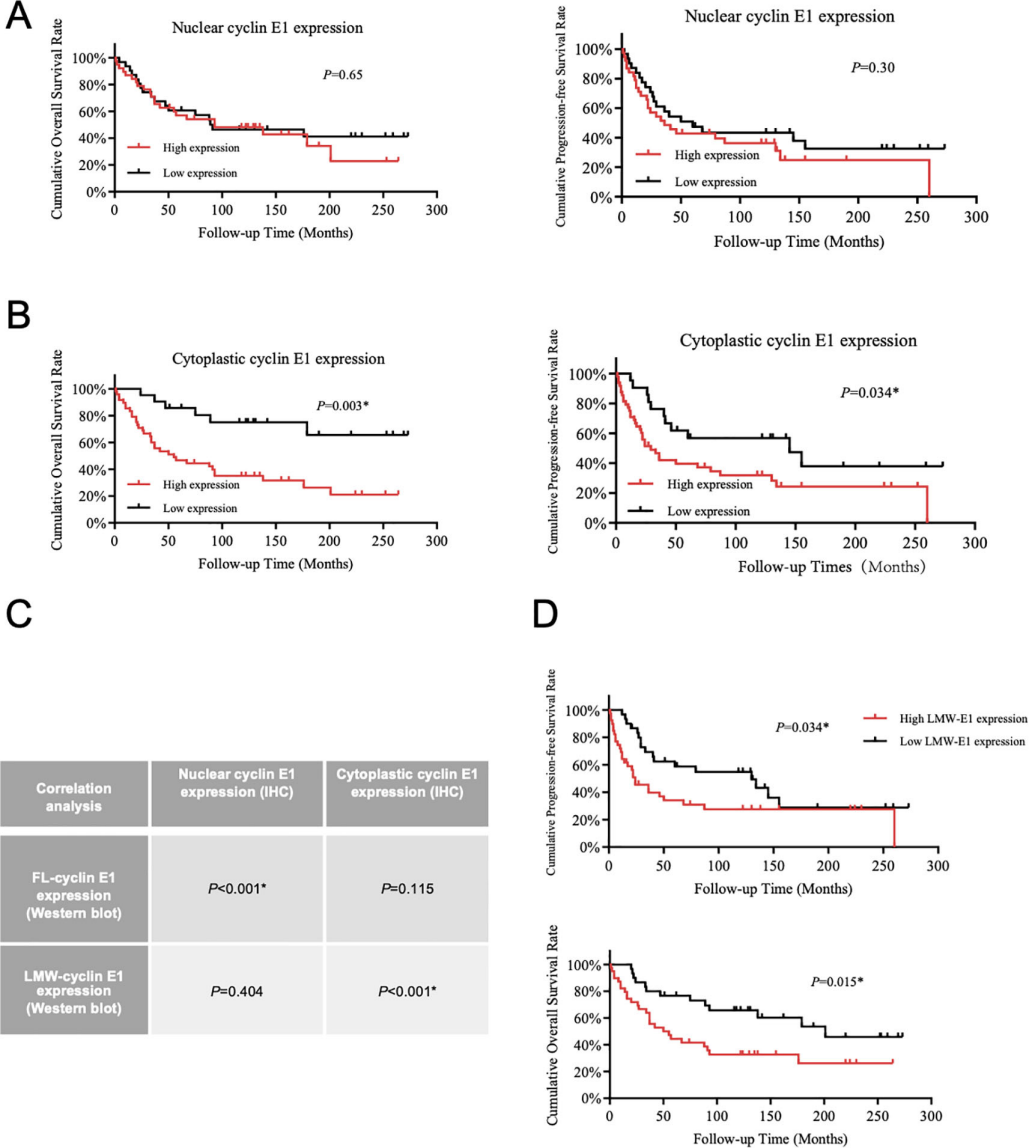
LMW-cyclin E1 was located in the cellular cytoplasm and was associated with poor outcomes in osteosarcoma patients. **(A)** The relationship between nuclear cyclin E1 expression and the overall survival rate and progression-free survival rate of osteosarcoma patients (Kaplan–Meier survival analysis). **(B)** The correlation analysis between cellular cytoplasmic cyclin E1 expression and the overall survival rate and progression-free survival rate of osteosarcoma patients (Kaplan–Meier survival analysis). **(C)** The correlation between the expression levels of different cyclin E1 isoforms and cyclin E1 at different subcellular sites in osteosarcoma tissue specimens. The results indicated that LMW-cyclin E1 was significantly correlated with cyclin E1 expression in the cellular cytoplasm (Chi-square test). **(D)** Quantitative analysis of the Western blot results showed that higher LMW-cyclin E1 expression was correlated with shorter overall survival and progression-free survival (Kaplan–Meier survival analysis). (*P<0.05, **P<0.01).

According to the cyclin E1 staining intensity in osteosarcoma tissue microarray, four staining patterns (staining score = 0, 1+, 2+, 3+) were defined separately for the cellular nucleus and cellular cytoplasm (**Figure 1B**). OS samples with staining scores of 0 and 1+ were classified into the low cyclin E1 expression group, whereas those with scores of 2+ and 3+ were classified into the high expression group. Correlation analysis revealed that only the cytoplasmic cyclin E1 expression level was significantly associated with the prognosis of osteosarcoma patients, while the nuclear cyclin E1 expression level was significantly associated with the prognosis of osteosarcoma patients, while the nuclear cyclin E1 expression level was not related to overall survival or progression-free survival (**Figures 2A, B**). These findings suggest that cytoplasmic cyclin E1 represents a distinct cyclin E1 subtype that may be more malignant in driving osteosarcoma progression.

As the presence of a subtype of LMW-cyclin E1 in osteosarcoma was shown at 33–47 kDa in Western blot assay (**Figure 1D**), we analyzed the expression level of LMW-cyclin E1 in relation to patient prognosis, and the results showed that the expression of LMW-cyclin E1 was significantly correlated with the overall survival and progression-free survival of osteosarcoma patients (**Figure 2D**). By integrating the immunohistochemical staining data and Western blot results, we further analyzed the correlation between the expression of the LMW-cyclin E1 subtype and the expression of cyclin E1 in the cytoplasm. The results showed that the expression level of cyclin E1 in the cytoplasm was significantly correlated with the expression of LMW-cyclin E1, whereas the expression level of cyclin E1 in the nucleus was not correlated with the expression of LMW-cyclin E1 (**Figure 2C**). Thus, these results indicated that LMW-cyclin E1 is located in the cytoplasm of osteosarcoma cells and is a specific subtype of cyclin E1 that is significantly associated with the prognosis of osteosarcoma.

### Investigate the proteases regulating LMW-cyclin E1 formation

**Figure 3:**
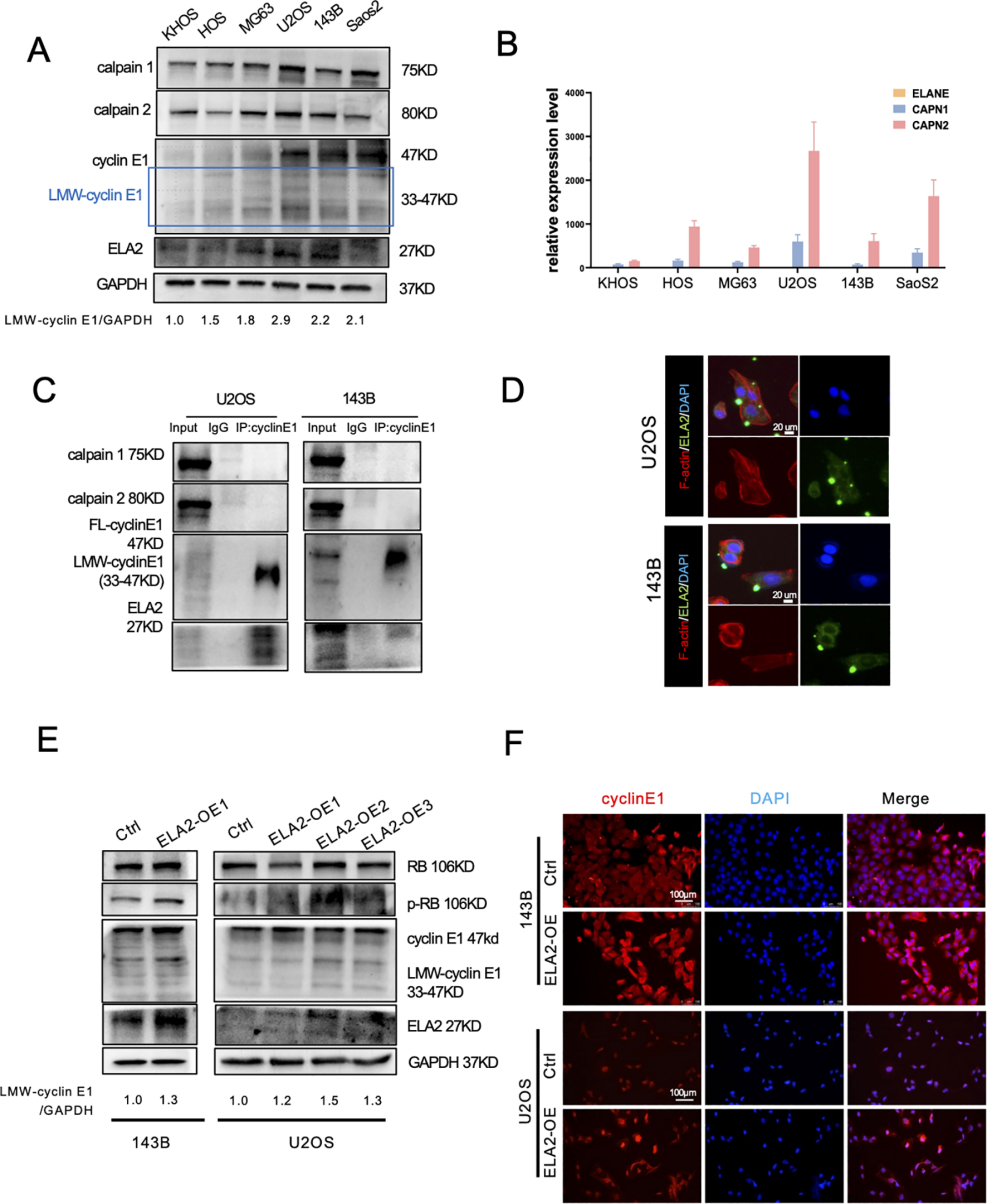
Calpain 1 and calpain 2 regulated FL-cyclin E1 expression, and neutrophil elastase (ELA2) accelerated LMW-cyclin E1 formation. **(A)** Western blot analysis of cyclin E1 (FL and LMW), calpain 1, calpain 2, and ELA2 expression in six OS cell lines. GAPDH served as the loading control (the assay was replicated three times). **(B)** Quantitative analysis of the relative mRNA expression levels of ELANE, CAPN1, and CAPN2 in OS cell lines by qPCR assay. **(C)** The interaction relationships between cyclin E1 and ELA2, and calpain 1 and calpain 2 were examined by IP–Western blot assays in U2OS and 143B (the assay was replicated three times). **(D)** Immunofluorescence staining showed that ELA2 was expressed in U2OS and 143B cell lines and could be secreted outside the cells (green: ELA2; red: F-actin; blue: DAPI; scale bar: 20 μm). **(E)** The protein expression of cyclin E1 (FL and LMW) and its downstream proteins (RB and p-RB) was assessed by Western blot after ELA2-OE plasmid transfection in U2OS and 143B cells (the assay was replicated three times). **(F)** Immunofluorescence images showed cyclin E1 expression after ELA2-OE plasmid transfection. Increased ELA2 upregulated cellular cytoplasmic cyclin E (Rred: cyclin E1; blue: DAPI; scale bar: 100 μm). (FL, full length; LMW, low molecular weight; p-RB, phosphorylated RB).

Many studies have revealed that several proteases can bind to FL-cyclin E1 to induce the formation of LMW-cyclin E1. Calpain 1 and calpain 2 are classical isoforms of the calpain family of calcium (Ca^2+^)-dependent cysteine proteases, and neutrophil elastase (ELA2) is a serine protease predominantly expressed in primary neutrophils; both have been reported to increase LMW-cyclin E1 expression (4, 19). We first detected the protein expression of cyclin E1, calpain 1, calpain 2, and ELA2 by Western blot assay and analyzed the corresponding mRNA levels (CCNE1, CAPN1, CAPN2, ELANE) via RT-PCR in OS cell lines (KHOS, HOS, MG63, U2OS, 143B, SaoS2). LMW-cyclin E1 was expressed in all OS cell lines, especially in 143B cells and U2OS cells (**Figure 3A**), and CCNE1 was shown with the highest expression in U2OS cells (**Supplementary Figure S2**). Calpain 1, calpain 2, and ELA2 were also present in all OS cells we tested. Considering the qPCR results, we found that calpain 1 and calpain 2 had significantly higher expression levels than ELA2 in each OS cell line (**Figures 3A, B**; **Supplementary Figure S2**).

To investigate the proteases that promote LMW-cyclin E1 formation, we performed a co-immunoprecipitation (Co-IP) assay to evaluate cyclin E1 binding proteins. The results suggested that using the cyclin E1 antibody can enrich the ELA2 but not calpain 1 or calpain 2. It revealed ELA2 may play a regulatory role for cyclin E through interacting with cyclin E1, but calpain 1 and calpain 2 show no direct interaction with cyclin E1 (**Figure 3C**).

For exploring the functions of calpain 1 and calpain 2, small interfering RNA (siRNA) was designed to decrease calpain 1, calpain 2 expression in OS cells, and the knockdown efficiency was validated by PCR assay and western blot (**Supplementary Figures S3A, B**; **Supplementary Table S2**). In U2OS cell lines, we found that knocking down calpain 1 and calpain 2 down-regulated the expression of FL-cyclin E1 and LMW-cyclin E1, so that it also inhibited RB phosphorylation, which is the downstream signal pathway for cyclin E1 (**Supplementary Figure S3C**). Immunofluorescence (IF) staining also showed FL-cyclin E1 and cellular cytoplasmic cyclin E1 (LMW-cyclin E1) were both decreased (**Supplementary Figure S3D**). However, calpain 1 and calpain 2 seem to play a more sophisticated role in osteosarcoma, which is shown with the opposite effects in 143B cell lines (**Supplementary Figures S4A–C**).

At the least, the above results revealed that calpain 1 and calpain 2 were not combined with cyclin E1; they may be the upstream molecules of cyclin E1 to regulate cyclin E1 expression, but not the LMW-cyclin E1 formation promoter. The regulation mechanism of the calpain family in osteosarcoma is worth further investigation.

### Neutrophil elastase induced LMW-cylcinE1 formation

Recent research has suggested that ELA2 is mainly released by tumor-associated neutrophils, but its expression and release from tumor cells remain uncertain. We found ELA2 was expressed in all OS cell lines (**Figures 3A, B**), and that using the cyclin E1 antibody can enrich the ELA2, indicating that ELA2 may play a regulatory role for cyclin E through direct interaction (**Figure 3C**). Using IF staining, we further validated that ELA2 was present and could be secreted in 143B and U2OS cells (**Figure 3D**). To investigate ELA2 function in OS, we overexpressed ELA2 using plasmid transfection. qPCR results showed ELA2 was significantly upregulated (**Supplementary Figure S4D**), along with the LMW-cyclin E1 upexpression and downstream RB phosphorylation (**Figure 3E**). The immunofluorescence staining results also showed that the cytoplasmic cyclin E1 was up-regulated after ELA2 overexpressed in OS cells (**Figure 3F**).

### Neutrophil elastase accelerated osteosarcoma cells’ proliferation *in vitro* and *in vivo*

**Figure 4:**
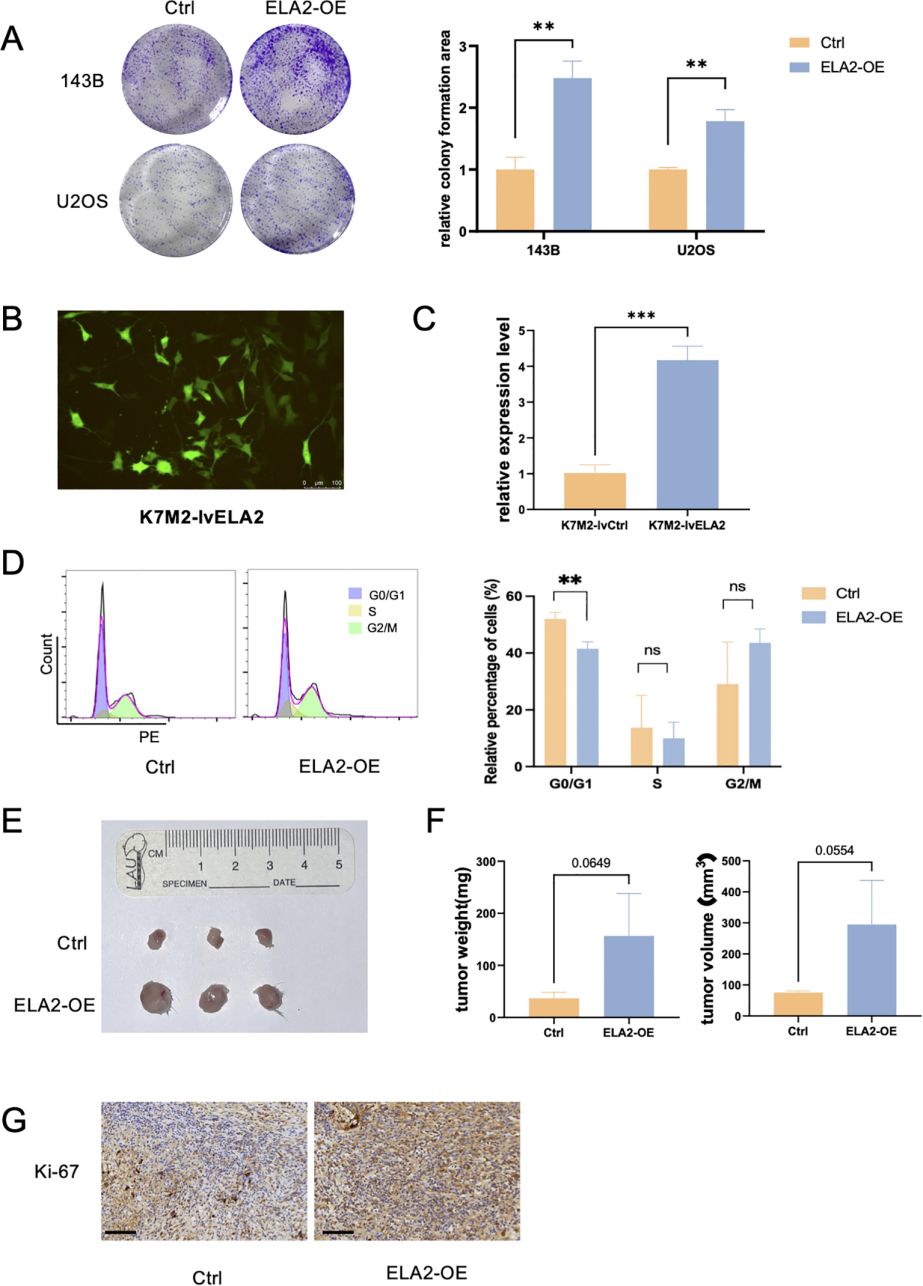
Osteosarcoma neutrophil elastase (ELA2) promotes osteosarcoma cell proliferation *in vitro* and *in vivo*. **(A)** Colony formation assay showed that ELA2 accelerated osteosarcoma proliferation in U2OS and 143B cells (unpaired two-tailed Student’s *t*-test, *N* = 3). **(B)** Immunofluorescence image showed that Lv-ELA2 was successfully transfected into K7M2 cell lines and stably expressed (scale bar: 100 μm). **(C)** Quantitative analysis of relative ELA2 expression level in the Lv-Ctrl group and Lv-ELA2 group by qPCR assays (unpaired two-tailed Student’s *t*-test, *N* = 3). **(D)** The results of the cell cycle in the Ctrl group and ELA2-OE group using flow cytometry (left). The statistical analysis of G0/G1 phase, S phase, and G2/M phase cell proportions (right) (unpaired two-tailed Student’s *t*-test, *N* = 3). **(E)** Images of tumors from the Ctrl group and ELA2-OE group tumors (*N* = 3 for each group). **(F)** Tumor weight and tumor volume were measured and quantitatively analyzed (tumor volume = length × width^2^/2; unpaired two-tailed Student’s *t*-test, *N* = 3). **(G)** Images of Ki-67^+^ immunohistochemistry staining in the Ctrl group and ELA2-OE group (scale bar: 100 μm). (^***^ *p <* 0.001).

Cyclin E1 is a cell cycle regulation protein that plays an important role in the G1/S transition. Thus, we further validated whether ELA2 can accelerate osteosarcoma proliferation. The results of the colony formation assay indicated that ELA2 can promote proliferative viability in OS cells (**Figure 4A**). In the mouse osteosarcoma cell line, K7M2-lvCtrl and K7M2-lvELA2 cells were constructed by lentiviral transfection. The transfection efficiency was validated under the fluorescence microscope and qPCR assay to examine the ELA2 mRNA expression, as we can see ELA2 was remarkably overexpressed (**Figures 4B, C**). The cell cycle assay was performed to validate the function of ELA2. The proportion of cells in the G0/G1 phase decreased significantly in the ELA2 overexpression group, indicating accelerated G1/S transition (**Figure 4D**). *In vivo*, BALB/c mice were used as models of subcutaneous tumor-bearing animals. After 25-day tumor bearing, the mice were sacrificed, and the tumor masses were removed for measurement. The tumor weight and tumor volume were obviously increased in the ELA2-OE group (**Figures 4E, F**). In addition, we performed Ki-67 immunohistochemical staining on xenograft tumor tissue, and the results showed that ELA2 overexpression increased Ki-67^+^ cells compared to the control group (**Figure 4G**), mirroring our *in vitro* data and confirming potent promotion of tumor cell proliferation.

### Neutrophils released ELA2 to induce LMW-cyclin E1 formation.

**Figure 5:**
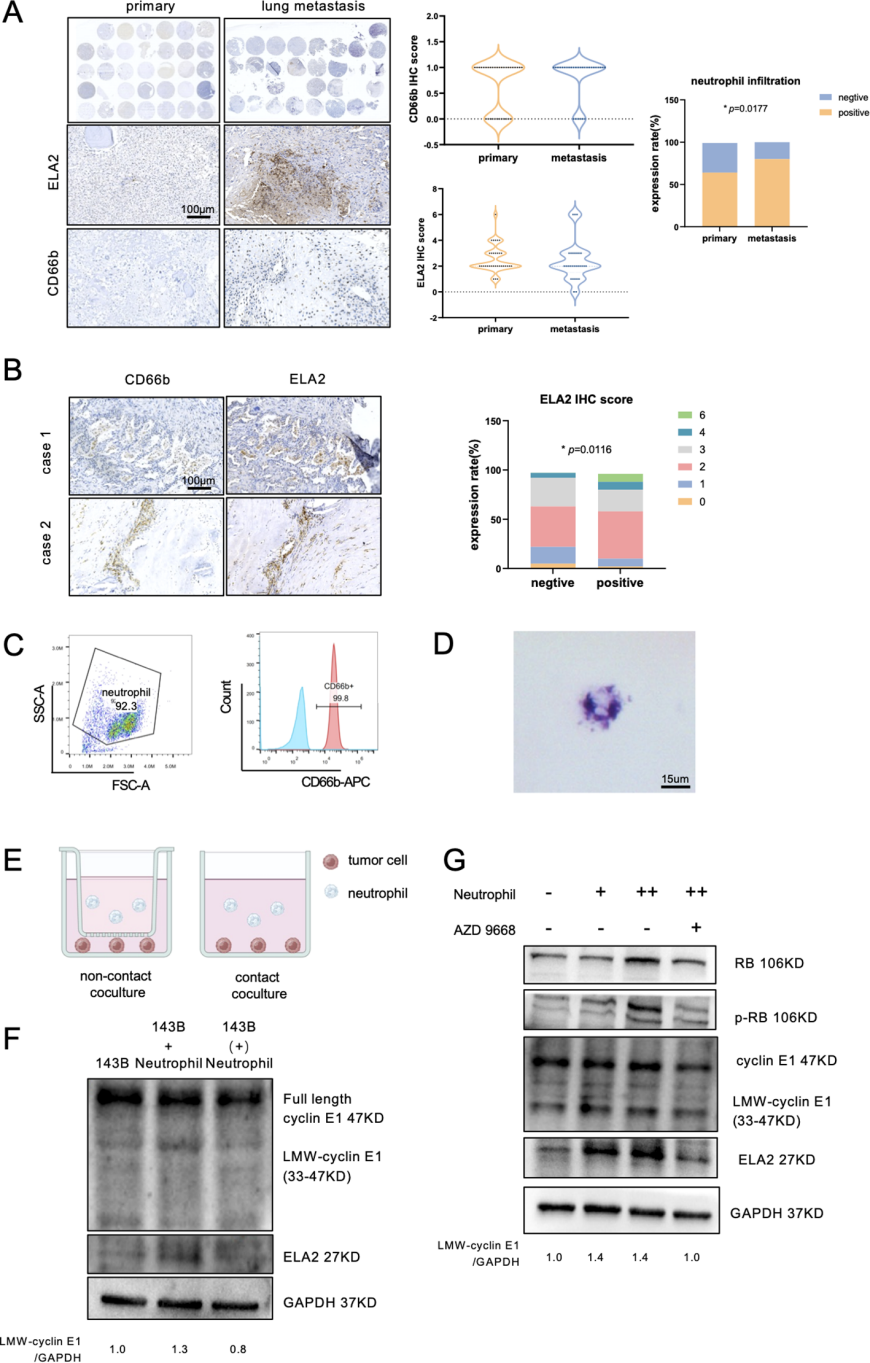
Neutrophils released ELA2 to induce LMW-cyclin E1 formation. **(A)** The expression of ELA2 and the infiltration of neutrophils in primary osteosarcoma tissues (*N* = 34) and lung metastasis tissues (*N* = 33) were assessed using IHC staining. The IHC scores of ELA2 and CD66b in the primary osteosarcoma group and the lung metastasis group are shown. Positive neutrophil infiltration was correlated with osteosarcoma lung metastasis (Fisher’s exact test; scale bar: 100 μm). **(B)** IHC staining images of CD66b and ELA2 at the same location in two patients. The analysis of the relationship between neutrophils and ELA2 revealed that greater neutrophil infiltration indicated higher ELA2 expression (Chi-square test; scale bar: 100 μm). **(C)** Flow cytometry was used to examine the purity of isolated neutrophils, with CD66b^+^ cells gated as neutrophils. **(D)** Giemsa staining was performed to observe the morphology of the isolated neutrophils, which exhibited multilobed, dark purple nuclei (scale bar: 15 μm). **(E)** Schematic diagram illustrating the contact coculture and noncontact coculture systems. **(F)** Western blot analysis of cyclin E1 (FL, LMW) and ELA2 expression after neutrophil contact or noncontact coculture with osteosarcoma (the assay was replicated three times). **(G)** The results showed that the expressions of ELA2 and LMW-cyclin E in osteosarcoma were upregulated and increased downstream RB phosphorylation by neutrophils. AZD9668 could reverse the ELA2 effect on osteosarcoma (the assay was replicated three times). (^*^ *p <* 0.05).

ELA2 was mainly secreted by tumor-associated neutrophils in various tumor types (20, 21). In osteosarcoma, the function was unclear, and whether neutrophil-derived ELA2 has a parallel effect with osteosarcoma-derived ELA2 was still uncertain. First, we performed IHC staining to observe neutrophil infiltration and ELA2 expression in 34 primary osteosarcoma tissue microarrays and 33 lung metastasis tissue microarrays. The results showed that neutrophil infiltration was slightly higher in primary osteosarcoma, but higher infiltration was seen in lung metastasis osteosarcoma samples (**Figure 5A**). According to the classical IHC staining score, which involved the staining intensity and area, IHC staining of CD66b and ELA2 was scored, and the samples of CD66b staining were all distributed in the 0 and 1 score groups. Considering the few neutrophil infiltrations, more than three neutrophil infiltrations in one field were defined as neutrophil-positive infiltration, and we found that neutrophil-positive infiltration was correlated with osteosarcoma lung metastasis (**Figure 5A**). Remarkably, neutrophil infiltration and ELA2 expression were shown at the same location, and neutrophil-positive infiltration was correlated with more ELA2 expression (**Figure 5B**).

Using density gradient centrifugation, we isolated neutrophils from the peripheral blood of healthy donors, and the purity was validated by flow cytometry (**Figure 5C**). The results of Giemsa stain also showed that the isolated neutrophils had multinuclei and were dark purple, which is the typical neutrophil feature (**Figure 5D**). To investigate neutrophils’ effect on osteosarcoma, 5 × 10^5^ neutrophils were cocultured with 5 × 10^5^ osteosarcoma cells in contact and noncontact ways for 24 h (**Figure 5E**). After 24-h coculture, the osteosarcoma cells’ total proteins were collected and detected by Western blot assay. Contacting coculture with neutrophils increased osteosarcoma ELA2 expression and promoted LMW-cyclinE1 expression. No obvious expression change was observed in noncontacted coculture with neutrophils (**Figure 5F**). To further validate the effect of neutrophil-derived ELA2 on osteosarcoma, AZD9668, a highly selective inhibitor of ELA2, was used. AZD9668 showed no toxicity for osteosarcoma cells from 1 to 128 nM (**Supplementary Figure S5**); 50 nM AZD9668 was selected for further experiments. Osteosarcoma cells were cocultured with different concentrations of neutrophils. As the concentration increased, the expressions of ELA2 and LMW-cyclin E1 were upregulated, promoting downstream RB phosphorylation. At the same time, AZD9668 could inhibit ELA2 to reverse neutrophils’ effect on osteosarcoma (**Figure 5G**).

### Osteosarcoma may induce neutrophil extracellular-trap formation to promote OS progression

**Figure 6:**
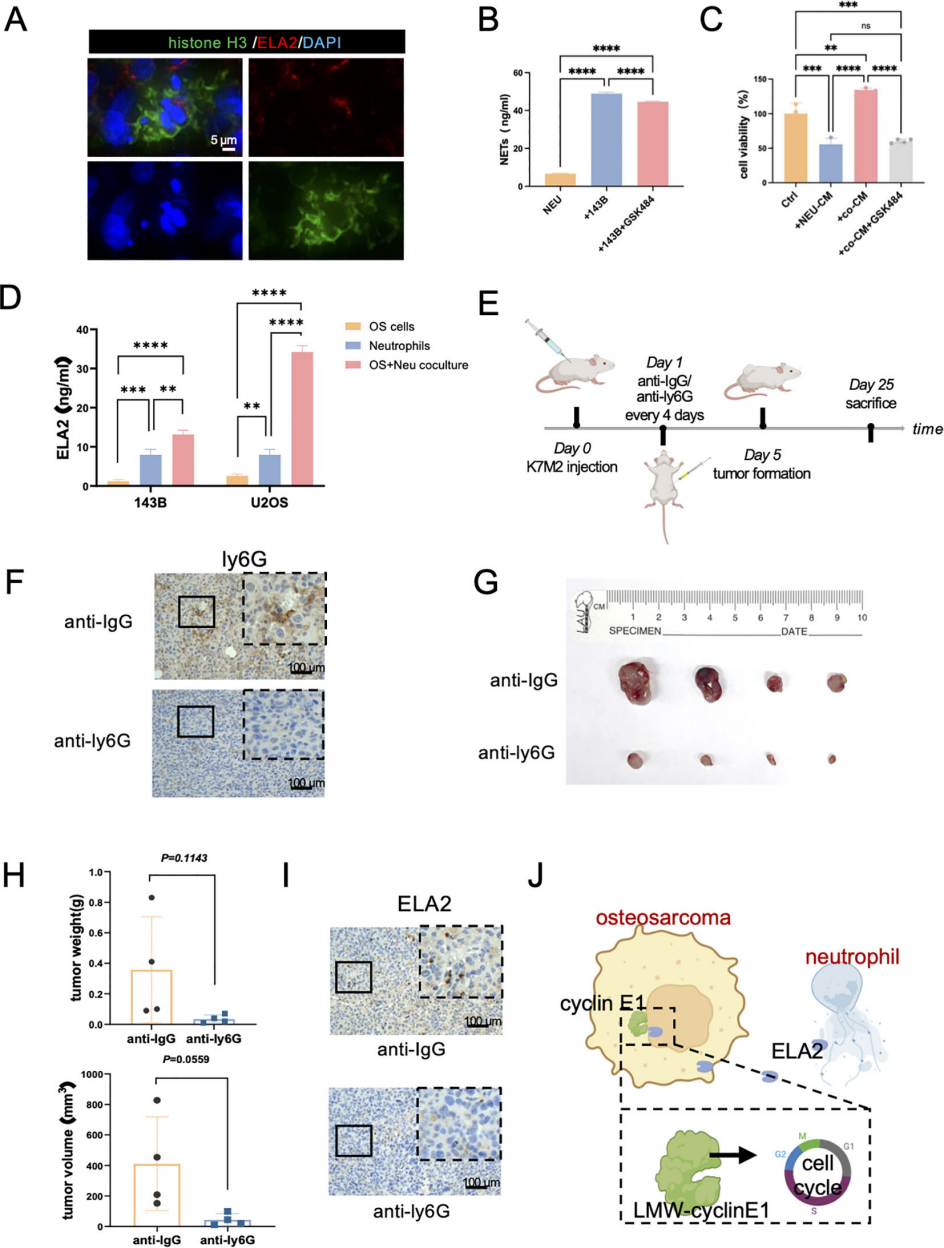
Osteosarcoma induces neutrophil extracellular traps (NETs) to promote OS progression. **(A)** Immunofluorescence staining results showed neutrophil extracellular traps in osteosarcoma tissues (green: Histone H3; red: ELA2; blue: DAPI; scale bar: 5 μm). **(B)** NET concentrations were tested in neutrophils, coculture system, and coculture with GSK484 by ELISA assay (one-way ANOVA test, *N* = 3 for each group). **(C)** The results of OS cell viability in the Ctrl group, neutrophil conditional medium group (NEU-CM), cocultured system conditional medium (co-CM), and cocultured system with GSK484 conditional medium (co-CM+GSK484) (one-way ANOVA test, *N* = 3 for each group). **(D)** ELA2 expression was tested in osteosarcoma cells, neutrophils, and a coculture system by ELISA assay (one-way ANOVA test, *N* = 3 for each group). **(E)** The schematic diagram of constructing tumor-bearing Balb/c mice and anti-ly6G intraperitoneal injection administration to exhaust neutrophils. **(F)** After anti-ly6G administration, IHC staining results indicated neutrophils were obviously decreased (*N* = 4 for each group; scale bar: 100 μm). **(G)** Images of osteosarcoma tissues from the anti-IgG group and the anti-Ly6G group. **(H)** Tumor weight and tumor volume were measured and quantitatively analyzed (tumor volume = length × width^2^/2; unpaired two-tailed Student’s *t*-test, *N* = 4 for each group). **(I)** Results of ELA2 IHC staining showed that anti-ly6G significantly inhibited ELA2 expression (scale bar: 100 μm). **(J)** The schematic diagram of our research shows that osteosarcoma induced neutrophil extracellular trap formation to secrete ELA2, which was taken up by osteosarcoma and promoted LMW-cyclin E1 formation, accelerating tumor progression (**P<0.01, ***P<0.001, ****P<0.0001).

NETs are web-like structures composed of granule proteins and decondensed chromatin, which are induced by surgical stress or inflammation. It is currently assumed that ELA2 is one of the main components of NETs. First, we verified the presence of NETs in osteosarcoma by immunofluorescence staining, showing that histone H3 was released into the extracellular space along with ELA2 (**Figure 6A**). In addition, the concentration of NETs in individually cultured neutrophils was 6.649 ng/ml. After coculturing with OS cells, NET formation was significantly increased, with the concentration rising to 48.90 ng/ml. Inhibiting NET formation with GSK484 also decreased the NET concentration to 44.61 ng/ml in the neutrophil-OS cell coculture system (**Figure 6B**). The results further revealed that OS can induce NET formation, and that this can be reversed by GSK484. To validate whether NETs could promote OS proliferation, we collected the conditioned medium of individually cultured neutrophils, cocultured neutrophils, and cocultured neutrophils treated with GSK484, and added them to the OS cell medium. As the results show, the conditioned medium of individually cultured neutrophils decreased the OS cells. However, after coculturing with OS cells, the neutrophils were educated into tumor-associated neutrophils, and NETs were formed. The conditioned medium of the coculture system could promote OS cell proliferation, and this effect could be inhibited by the NET inhibitor GSK484 (**Figure 6C**).

As for ELA2 expression, we also performed the ELISA assay to test the ELA2 concentrations in individually cultured neutrophils and the cocultured system. As the results show, after coculture with OS cells, ELA2 expression was significantly increased in the coculture system, which also indicated NETs were formed and triggered ELA2 release (**Figure 6D**). At the same time, the results of the ELISA assay also supported our previous findings that neutrophils had higher expression of ELA2 than OS cells, which were the main source of ELA2 in osteosarcoma.

We further investigated the functions of neutrophils in osteosarcoma malignant progression *in vivo*. Balb/c mice were used to construct a subcutaneous osteosarcoma model, and anti-ly6G was administered via intraperitoneal injection every 4 days to exhaust neutrophils. After 25 days of treatment, the tumor tissues were removed, and the IHC results showed the neutrophils were significantly exhausted after anti-Ly6G treatment (**Figures 6E, F**). Remarkably, neutrophil exhaustion obviously hindered osteosarcoma progression; both tumor weight and volume were significantly inhibited (**Figures 6G, H**). In addition, the lungs of mice were harvested for histological examination. In the anti-ly6G group, there was no lung metastasis. In the Ctrl group, the lung metastasis niche was observed (**Supplementary Figure S6**). While the results did not reach statistical significance, we observed a trend suggesting that neutrophil depletion may reduce lung metastasis formation. After depleting neutrophils, the expression level of ELA2 was also significantly deregulated (**Figure 6I**). All the results indicated that targeting neutrophils and their ELA2 expression may be a prospective immunotherapy strategy for osteosarcoma (**Figure 6J**).

## Discussion

Osteosarcoma is the most common primary malignant bone tumor in adolescents and young adults, characterized by highly aggressive progression due to its unrestricted proliferative capability. Cyclin E1, an essential cell cycle-regulating protein that binds the CDK2, forms the active CDK2-cyclin E holoenzyme to regulate the G1/S phase transition. Dysregulation of cyclin E has been reported in many solid tumors. In our previous study, we found that cyclin E1 was significantly overexpressed in osteosarcoma, and its expression was positively correlated with disease status and inversely correlated with prognosis and chemotherapeutic response (11). However, the discrepant effects on osteosarcoma patients’ prognosis between CCNE1 and cyclin E1 protein indicated that there may be a hyperactive subtype of cyclin E1. In several types of solid malignancies, LMW-cyclin E1 was reported as a more hyperactive isoform than full-length cyclin E1, resulting in deregulation of the G1 to S transition. LMW-cyclin E1 can maintain the viability of cancer cells by facilitating replication stress tolerance (7) and regulating lipid metabolism (22). However, whether LMW-cyclin E1 is present in osteosarcoma remains unclear. Thus, in this study, we first revealed that LMW-cyclin E1 isoforms are expressed and localized in the cytoplasm of osteosarcoma using a series of assays. LMW-cyclin E1 was more strongly associated with poor prognosis of osteosarcoma patients than FL-cyclin E1, further demonstrating the critical role of LMW-cyclin E1 in promoting malignant biological behavior.

The formation mechanisms and functions of LMW-cyclin E1 vary across different cancer types. It has been reported that calpain and ELA2 can cleave cyclin E1, resulting in LMW forms of cyclin E in breast cancer (19). Cyclin E1 can regulate and be regulated by calpain 2, as cyclin E1 increases calpain 2 transcription, leading to LMW-cyclin E1 formation (23). In our study, the expression of calpain 1 and calpain 2 was first examined, and the results showed that calpain family proteins were clearly present in all osteosarcoma cell lines. However, as the Western blot results indicated, calpain regulated FL-cyclin E1 expression but not cyclin E1 cleavage, which differs from the modification mechanism in breast cancer. Interestingly, calpain appears to have diverse functions in different OS cell lines. Considering the high heterogeneity of osteosarcoma, the calpain family may act as upstream regulators of cyclin E1. Our data reveal opposing effects of calpain 1 and calpain 2 knockdown in U2OS versus 143B cells. This divergence likely derived from intrinsic biological differences between these cells. U2OS is a telomerase-negative cell that depends on the alternative lengthening of telomeres (ALT) pathway, rendering it reliant on calpain-mediated cyclin E1 cleavage for DNA damage adaptation (24, 25). Conversely, 143B is telomerase positive, and its telomerase activity allows calpain loss while priming DNA repair (26). These findings underscore that osteosarcoma subtypes utilize calpains in context-dependent manners. In telomerase-positive tumors (like 143B), calpain inhibition may inadvertently support cyclin E1-driven progression, whereas in ALT-dependent tumors (like U2OS), it exacerbates genomic instability. Future therapies targeting calpains should consider subtype stratification based on telomere maintenance.

ELA2, the main granzyme ingredient of neutrophils, is generally acknowledged to be secreted by neutrophils, with few studies demonstrating its expression in tumor cells. In our research, we first demonstrated that ELA2 is also expressed in osteosarcoma cells and can be secreted into extracellular media, broadening the recognized expression profile of ELA2. Our results suggest that ELA2 is naturally present in OS cells at low levels, and upregulated ELA2 in osteosarcoma accelerates LMW-cyclin E1 formation. Meanwhile, it is indisputable that neutrophils remain the primary source of ELA2; coculture with neutrophils stimulates increased ELA2 release, and uptake of neutrophil-derived ELA2 further enhances LMW-cyclin E1 formation.

Neutrophils are the most abundant white blood cells in circulation, traditionally recognized as immune defense cells that play an irreplaceable role in protecting the body against pathogen invasion through phagocytosis and degranulation (27). Paradoxically, recent findings suggest that neutrophils also play crucial roles during carcinogenesis. They can crosstalk with other immune cells, such as inhibiting DCs’ antigen presentation and impairing T-cell function (28, 29). Additionally, neutrophils can create a supportive environment that promotes tumor initiation, proliferation, metastasis, and angiogenesis (30). In osteosarcoma, recent findings have shown that neutrophils participate in the pulmonary metastasis process. High neutrophil infiltration contributes to the formation of a premetastatic niche that facilitates the circulation of OS cells to lung metastasis (31, 32). NETs are web-like structures composed of neutrophil extracellular DNA and associated proteolytic enzymes, including ELA2. Traditionally regarded as pathogen-trapping structures, NETs are now increasingly recognized for their role in promoting tumor metastasis. In osteosarcoma, some researchers have developed a NET-related metastasis signature to predict metastatic recurrence in patients (33), which is also associated with patients’ immune statuses and drug sensitivity (34). However, further experiments to explore the functions of neutrophils and NETs in osteosarcoma remain essential. In our study, we found that neutrophil infiltration was higher in lung metastasis tissues and correlated with ELA2 expression. Depleting neutrophils in mice using Ly6G antibodies inhibited tumor proliferation, highlighting the significant potential of targeting neutrophils in OS immunotherapy. Additionally, immunofluorescence staining revealed the clear presence of NETs’ dual-positive network structures in osteosarcoma tissue.

However, we acknowledge several limitations in this research. The immunofluorescence assay may have produced false positives, so fluorescence intensity was not quantified. More objective and quantifiable experiments are needed to verify the presence of NETs in OS. The duration of tumor bearing in animal models was relatively short, and lung metastasis was not established, limiting our ability to assess neutrophils’ role in metastasis; thus, we only demonstrated their function in OS proliferation. In addition, although our *in vivo* data consistently show protumor effects of ELA2, the sample size remains a limitation. Future large-scale studies are required to validate these findings across diverse osteosarcoma models.

Overall, our data show that the regulation of LMW-cyclin E1 is driven by neutrophil elastase in osteosarcoma, and the crosstalk between osteosarcoma cells and neutrophils may offer a novel therapeutic strategy.

## Data Availability

The original contributions presented in the study are included in the article/material. Further inquiries can be directed to the corresponding authors.

## References

[1] Cole S Gianferante DM Zhu B Mirabello L. Osteosarcoma: A Surveillance, Epidemiology, and End Results program-based analysis from 1975 to 2017. Cancer. (2022) 128:2107–18. doi: 10.1002/cncr.34163, PMID: 35226758 PMC11647566

[2] Ritter J Bielack SS. Osteosarcoma. Ann oncology: Off J Eur Soc Med Oncol. (2010) 21 Suppl 7:vii320–5. doi: 10.1093/annonc/mdq276, PMID: 20943636

[3] Smeland S Bielack SS Whelan J Bernstein M Hogendoorn P Krailo MD. Survival and prognosis with osteosarcoma: outcomes in more than 2000 patients in the EURAMOS-1 (European and American Osteosarcoma Study) cohort. Eur J Cancer. (2019) 109:36–50. doi: 10.1016/j.ejca.2018.11.027, PMID: 30685685 PMC6506906

[4] Caruso JA Duong MT Carey JPW Hunt KK Keyomarsi K. Low-molecular-weight cyclin E in human cancer: cellular consequences and opportunities for targeted therapies. Cancer Res. (2018) 78:5481–91. doi: 10.1158/0008-5472.CAN-18-1235, PMID: 30194068 PMC6168358

[5] Karst AM Jones PM Vena N Ligon AH Liu JF Hirsch MS. Cyclin E1 deregulation occurs early in secretory cell transformation to promote formation of fallopian tube-derived high-grade serous ovarian cancers. Cancer Res. (2014) 74:1141–52. doi: 10.1158/0008-5472.CAN-13-2247, PMID: 24366882 PMC4517944

[6] Chu C Geng Y Zhou Y Sicinski P. Cyclin E in normal physiology and disease states. Trends Cell Biol. (2021) 31:732–46. doi: 10.1016/j.tcb.2021.05.001, PMID: 34052101 PMC8364501

[7] Li M Tsavachidis S Wang F Bui T Nguyen TDT Luo L. Low-molecular-weight cyclin E deregulates DNA replication and damage repair to promote genomic instability in breast cancer. Oncogene. (2022) 41:5331–46. doi: 10.1038/s41388-022-02527-z, PMID: 36344674 PMC9742291

[8] Lulla AR Akli S Karakas C Ha MJ Fowlkes NW Mitani Y. LMW cyclin E and its novel catalytic partner CDK5 are therapeutic targets and prognostic biomarkers in salivary gland cancers. Oncogenesis. (2021) 10:40. doi: 10.1038/s41389-021-00324-z, PMID: 33990543 PMC8121779

[9] Doostan I Karakas C Kohansal M Low KH Ellis MJ Olson JA Jr.. Cytoplasmic cyclin E mediates resistance to aromatase inhibitors in breast cancer. Clin Cancer Res. (2017) 23:7288–300. doi: 10.1158/1078-0432.CCR-17-1544, PMID: 28947566 PMC5768442

[10] Zhou YJ Xie YT Gu J Yan L Guan GX Liu X. Overexpression of cyclin E isoforms correlates with poor prognosis in rectal cancer. Eur J Surg oncology: J Eur Soc Surg Oncol Br Assoc Surg Oncol. (2011) 37:1078–84. doi: 10.1016/j.ejso.2011.08.139, PMID: 21944050

[11] Wei R Thanindratarn P Dean DC Hornicek FJ Guo W Duan Z. Cyclin E1 is a prognostic biomarker and potential therapeutic target in osteosarcoma. J orthopaedic research: Off Publ Orthopaedic Res Society. (2020) 38:1952–64. doi: 10.1002/jor.24659, PMID: 32162720

[12] Yang S Jia J Wang F Wang Y Fang Y Yang Y. Targeting neutrophils: Mechanism and advances in cancer therapy. Clin Transl Med. (2024) 14:e1599. doi: 10.1002/ctm2.1599, PMID: 38450975 PMC10918741

[13] Hedrick CC Malanchi I. Neutrophils in cancer: heterogeneous and multifaceted. Nat Rev Immunol. (2022) 22:173–87. doi: 10.1038/s41577-021-00571-6, PMID: 34230649

[14] Xue R Zhang Q Cao Q Kong R Xiang X Liu H. Liver tumour immune microenvironment subtypes and neutrophil heterogeneity. Nature. (2022) 612:141–7. doi: 10.1038/s41586-022-05400-x, PMID: 36352227

[15] Li L Li Y Lu M Wang Y Li Z Hu X. The combination of baseline neutrophil to lymphocyte ratio and dynamic changes during treatment can better predict the survival of osteosarcoma patients. Front Oncol. (2023) 13:1235158. doi: 10.3389/fonc.2023.1235158, PMID: 38033504 PMC10682781

[16] Boeltz S Amini P Anders HJ Andrade F Bilyy R Chatfield S. To NET or not to NET:current opinions and state of the science regarding the formation of neutrophil extracellular traps. Cell Death differentiation. (2019) 26:395–408. doi: 10.1038/s41418-018-0261-x, PMID: 30622307 PMC6370810

[17] Zeng W Song Y Wang R He R Wang T. Neutrophil elastase: From mechanisms to therapeutic potential. J Pharm analysis. (2023) 13:355–66. doi: 10.1016/j.jpha.2022.12.003, PMID: 37181292 PMC10173178

[18] Xu J Chen C Sun K Shi Q Wang B Huang Y. Tocilizumab (monoclonal anti-IL-6R antibody) reverses anlotinib resistance in osteosarcoma. Front Oncol. (2023) 13:1192472. doi: 10.3389/fonc.2023.1192472, PMID: 37404767 PMC10315670

[19] Wang XD Rosales JL Magliocco A Gnanakumar R Lee KY. Cyclin E in breast tumors is cleaved into its low molecular weight forms by calpain. Oncogene. (2003) 22:769–74. doi: 10.1038/sj.onc.1206166, PMID: 12569370

[20] Cui C Chakraborty K Tang XA Zhou G Schoenfelt KQ Becker KM. Neutrophil elastase selectively kills cancer cells and attenuates tumorigenesis. Cell. (2021) 184:3163–77.e21. doi: 10.1016/j.cell.2021.04.016, PMID: 33964209 PMC10712736

[21] Tan Q Ma X Yang B Liu Y Xie Y Wang X. Periodontitis pathogen Porphyromonas gingivalis promotes pancreatic tumorigenesis via neutrophil elastase from tumor-associated neutrophils. Gut Microbes. (2022) 14:2073785. doi: 10.1080/19490976.2022.2073785, PMID: 35549648 PMC9116393

[22] Lucenay KS Doostan I Karakas C Bui T Ding Z Mills GB. Cyclin E associates with the lipogenic enzyme ATP-citrate lyase to enable Malignant growth of breast cancer cells. Cancer Res. (2016) 76:2406–18. doi: 10.1158/0008-5472.CAN-15-1646, PMID: 26928812 PMC4873469

[23] Libertini SJ Robinson BS Dhillon NK Glick D George M Dandekar S. Cyclin E both regulates and is regulated by calpain 2, a protease associated with metastatic breast cancer phenotype. Cancer Res. (2005) 65:10700–8. doi: 10.1158/0008-5472.CAN-05-1666, PMID: 16322214

[24] Gurung RL Lim HK Venkatesan S Lee PS Hande MP. Targeting DNA-PKcs and telomerase in brain tumour cells. Mol Cancer. (2014) 13:232. doi: 10.1186/1476-4598-13-232, PMID: 25307264 PMC4213508

[25] Maciejewska N Olszewski M Jurasz J Baginski M Stasevych M Zvarych V. Teloxantron inhibits the processivity of telomerase with preferential DNA damage on telomeres. Cell Death Dis. (2022) 13:1005. doi: 10.1038/s41419-022-05443-y, PMID: 36437244 PMC9701690

[26] Hu Y Bobb D He J Hill DA Dome JS. The HSP90 inhibitor alvespimycin enhances the potency of telomerase inhibition by imetelstat in human osteosarcoma. Cancer Biol Ther. (2015) 16:949–57. doi: 10.1080/15384047.2015.1040964, PMID: 25920748 PMC4622625

[27] Liew PX Kubes P. The neutrophil’s role during health and disease. Physiol Rev. (2019) 99:1223–48. doi: 10.1152/physrev.00012.2018, PMID: 30758246

[28] Ugolini A Tyurin VA Tyurina YY Tcyganov EN Donthireddy L Kagan VE. Polymorphonuclear myeloid-derived suppressor cells limit antigen cross-presentation by dendritic cells in cancer. JCI Insight. (2020) 5:e138581. doi: 10.1172/jci.insight.138581, PMID: 32584791 PMC7455061

[29] Kim R Hashimoto A Markosyan N Tyurin VA Tyurina YY Kar G. Ferroptosis of tumour neutrophils causes immune suppression in cancer. Nature. (2022) 612:338–46. doi: 10.1038/s41586-022-05443-0, PMID: 36385526 PMC9875862

[30] Huang X Nepovimova E Adam V Sivak L Heger Z Valko M. Neutrophils in Cancer immunotherapy: friends or foes? Mol Cancer. (2024) 23:107. doi: 10.1186/s12943-024-02004-z, PMID: 38760815 PMC11102125

[31] Tang F Tie Y Lan TX Yang JY Hong WQ Chen SY. Surgical treatment of osteosarcoma induced distant pre-metastatic niche in lung to facilitate the colonization of circulating tumor cells. Advanced Sci (Weinheim Baden-Wurttemberg Germany). (2023) 10:e2207518. doi: 10.1002/advs.202207518, PMID: 37585564 PMC10558698

[32] Almeida SFF Santos L Sampaio-Ribeiro G Ferreira HRS Lima N Caetano R. Unveiling the role of osteosarcoma-derived secretome in premetastatic lung remodelling. J Exp Clin Cancer Res. (2023) 42:328. doi: 10.1186/s13046-023-02886-9, PMID: 38031171 PMC10688015

[33] Tang H Xie J Du YX Tan ZJ Liang ZT. Osteosarcoma neutrophil extracellular trap network-associated gene recurrence and metastasis model. J Cancer Res Clin Oncol. (2024) 150:48. doi: 10.1007/s00432-023-05577-2, PMID: 38285218 PMC10824883

[34] Lin Y Tang H Teng H Feng W Li F Liu S. Development and validation of neutrophil extracellular traps-derived signature to predict the prognosis for osteosarcoma patients. Int immunopharmacology. (2024) 127:111364. doi: 10.1016/j.intimp.2023.111364, PMID: 38101221

