# Supplementary material for: Neutrophil elastase promotes low molecular weight cyclin E1 formation to accelerate osteosarcoma proliferation
Source: Front Immunol. 2025 Sep 1;16:1647913. doi: 10.3389/fimmu.2025.1647913 (PMC12434046; doi:10.3389/fimmu.2025.1647913)
Supplement: Supplementary file 1 [file DataSheet1.pdf]

Neutrophil elastase promotes low molecular weight cyclin E1 formation to accelerate osteosarcoma proliferation

Jiuhui Xu[1,2], Qianyu Shi[1,2], Fanwei Zeng[1,2], Tingting Ren[1,2], Ran Wei[1,2]*, Xiaodong Tang[1,2]*

1 Department of Musculoskeletal Tumor, Peking University People's Hospital, Beijing, China.
2 Beijing Key Laboratory of Musculoskeletal Tumor, Peking University People's Hospital, Beijing, China.

Supplemental information contains 6 supplemental figures and 3 supplemental tables.

S1 Fig. The expression of cyclin E1 in hFOB 1.19 cell line.

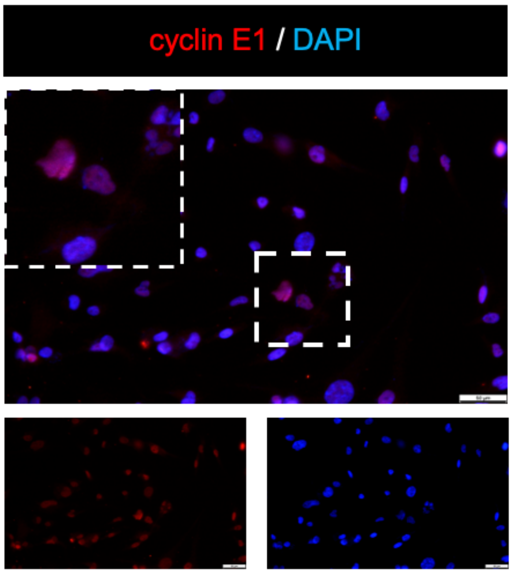

S2 Fig. The relative mRNA expression of CCNE1, CAPN1, CAPN2 and ELANE in osteosarcoma cell lines.(scale bar 50µm)

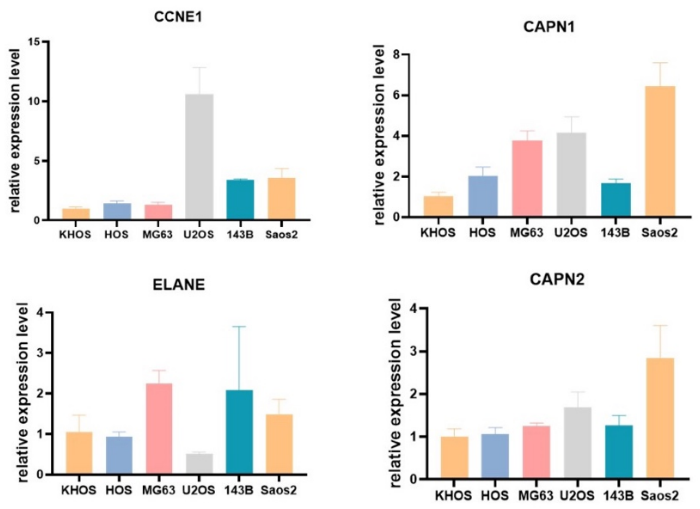

S3 Fig. Interfering calpain 1 and calpain 2 expression decreased FL-cyclin E1 and LMW-cyclin E1 expression in U2OS cell lines.

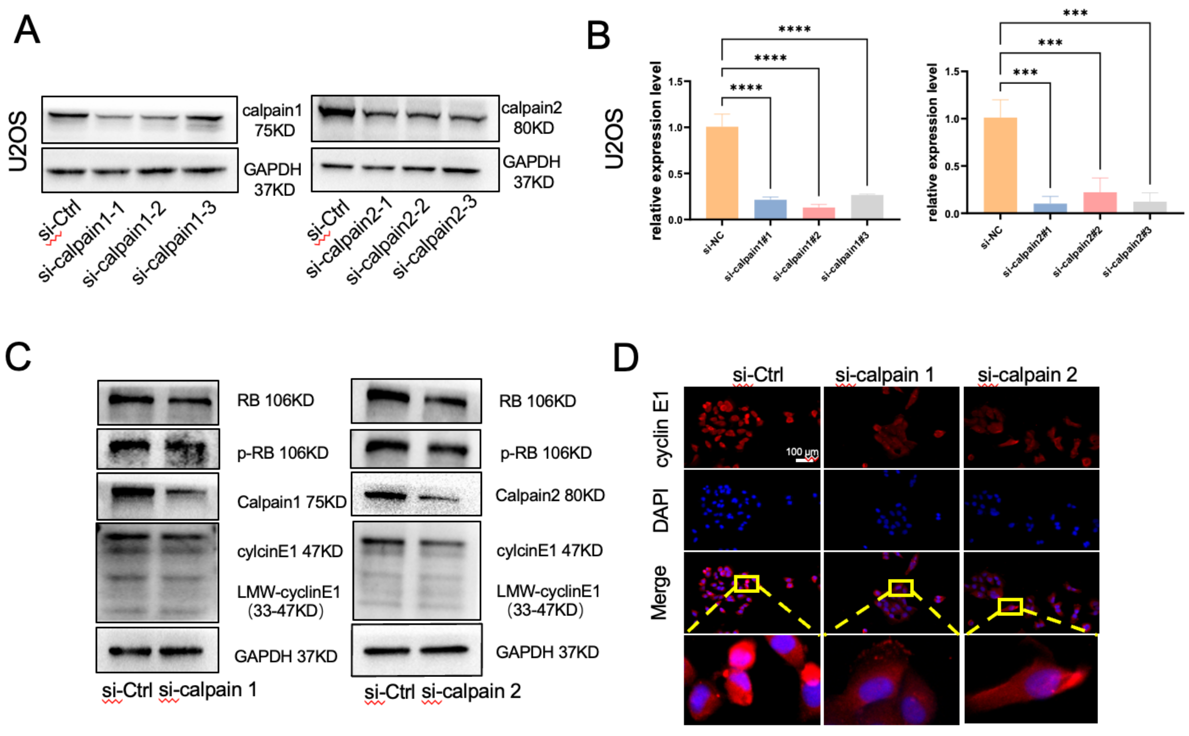

A. Western blot results showed calpain 1 and calpain 2 were obviously decreased in U2OS cell line (The assay was replicated three times).

B. The results of qPCR showed the three siRNA knock down efficiency in U2OS cell line (One-way ANOVA test).

C. The protein expression of cyclin E1 (FL and LMW) and its downstream proteins (RB and p-RB)

tested by Western blots after si-calpain 1 and si-calpain 2 transfection in U2OS cell line (The assay was replicated three times).

D. Immunofluorescence images showed cyclin E1 expression after si-calpain 1 and si-calpain 2 transfection in U2OS cell line. Knocking down calpain 1 and calpain 2 decreased cellular nucleus and cytoplasmic cyclin E1 (Red: cyclin E1, Blue: DAPI, scale bar 100μm).

(FL: full length, LMW: low molecular weight, p-RB: phosphorylated RB, ***$P < 0.001$, ****$P < 0.0001$)

S4 Fig. Interfering calpain 1 and calpain 2 expression increased FL-cyclin E1 and LMW-cyclin E1 expression which contributes to RB phosphorylation in 143B.

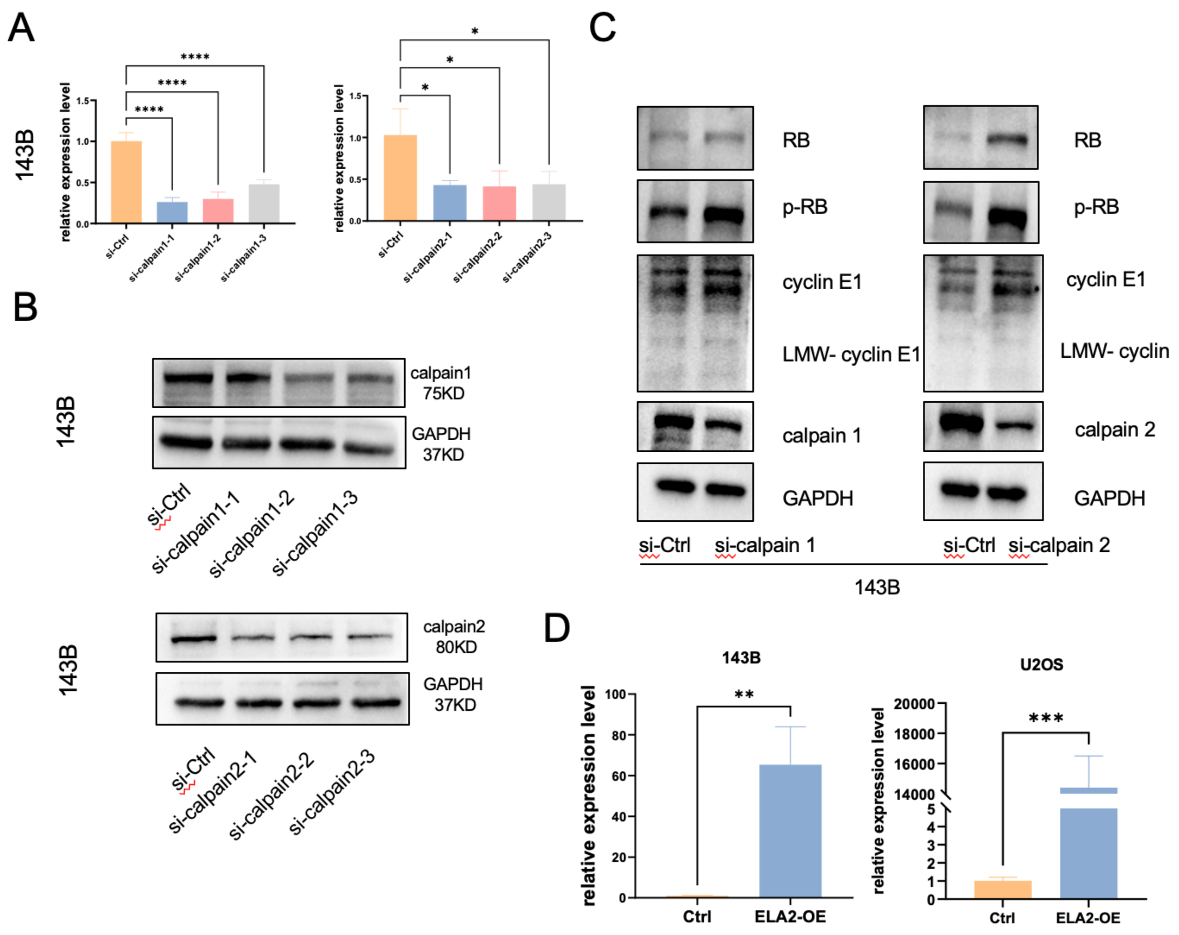

A. The results of qPCR showed the three siRNA knock down efficiency in 143B cell line (One-way ANOVA test).

B. Western blot results showed calpain 1 and calpain 2 were obviously decreased in 143B cell line (The assay was replicated three times).

C. The protein expression of cyclin E1 (FL and LMW) and its downstream proteins (RB and p-RB) tested by Western blots after si-calpain 1 and si-calpain 2 transfection in 143B cell line (The assay was replicated three times).

D. The results of qPCR showed the expression level of ELA2 was significantly upregulated by transfecting (Unpaired two-tailed Student t test).

S5 Fig. AZD9668 was shown with no toxicity for osteosarcoma cells from 1 to 128 nM (One-way ANOVA test).

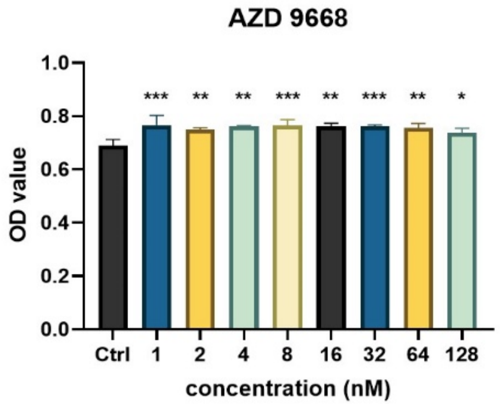

S6 Fig. H&E staining of mouse lung metastasis in anti-IgG group and anti-Ly6G group(scale bar 2.5 mm, 250μm).

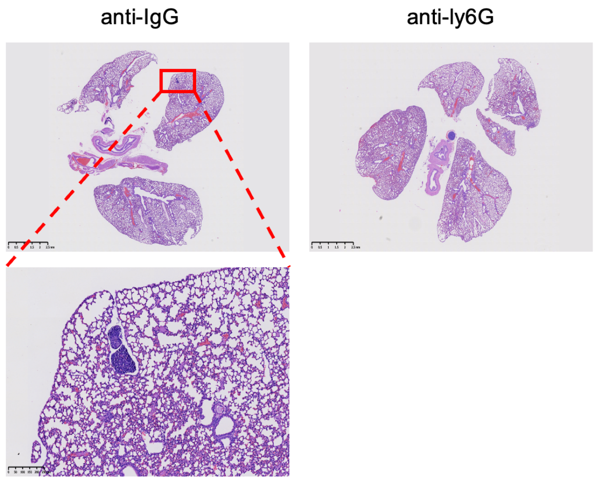

Supplementary Table 1. The primers for PCR assays

Gene	Forward	Reverse
ELANE	TGCGCCCAACTTCGTCATGTCG	CGTAGCCGTTTTCGAAGATGCG
CAPN1	GGTGGAGTTCAACATCCTGTGG	ATCCGCATCTCGTAGGCACTCA
CAPN2	AGGACATGCACACCATCGGCTT	CGGAGGTTGATGAAGGTGTCTG
CCNE1	TGTGTCCTGGATGTTGACTGCC	CTCTATGTCGCACCACTGATACC
GAPDH	GTCTCCTCTGACTTCAACAGCG	ACCACCCTGTTGCTGTAGCCAA

Supplementary Table 2. The sequences for siRNA

	Sense(5'-3')	Antisense(5'-3')
si-calpain1#1	GGAAGCUAGUGUUCGUGCATT	UGCACGAACACUAGCUUCCTT
si-calpain1#2	GCUCAGAGCAGUUCAUCAATT	UUGAUGAACUGCUCUGAGCTT
si-calpain1#3	GCUUCAAGCUCAACAAGAATT	UUCUUGUUGAGCUUGAAGCTT
si-calpain2#1	CCAGCGAUACCUACAAGAATT	UUCUUGUAGGUAUCGCUGGTT
si-calpain2#2	GCUCAGACACCUUCAUCAATT	UUGAUGAAGGUGUCUGAGCTT
si-calpain2#3	CGCUAUUCAAGAUAUUUAATT	UUAAAUAUCUUGAAUAGCGTT
si-negtive control	UUCUCCGAACGUGUCACGUTT	ACGUGACACGUUCGGAGAATT

Supplementary Table 3. The information of antibody

Antibody	Source	Identifier
cyclin E1	Abcam	ab133266
cyclin E1	Proteintech	11554-1-AP
calpain 1	Proteintech	10538-1-AP
calpain 2	Proteintech	11472-1-AP
ELA2	Abcam	ab131260
RB	Proteintech	10048-2-Ig
Rb (phospho S807)	Abcam	ab184796
GAPDH	Proteintech	60004-1-Ig
Histone H3	Abcam	ab176842
CD66b	Abcam	ab197678
CD66b	Yojanbio	E-AB-F1267E
anti-mouse Ly-6G	Biolegend	127649
